# Oncolytic Measles Virus Encoding MicroRNA for Targeted RNA Interference †

**DOI:** 10.3390/v15020308

**Published:** 2023-01-22

**Authors:** Sophie C. Anker, Marie G. Szczeponik, Jan Dessila, Katia Dittus, Christine E. Engeland, Dirk Jäger, Guy Ungerechts, Mathias F. Leber

**Affiliations:** 1 Clinical Cooperation Unit Virotherapy, German Cancer Research Center (DKFZ), Im Neuenheimer Feld 280, 69120 Heidelberg, Germany; 2 Department of Internal Medicine I and Clinical Chemistry, Heidelberg University Hospital, Im Neuenheimer Feld 671, 69120 Heidelberg, Germany; 3 Medical School, Heidelberg University, Im Neuenheimer Feld 672, 69120 Heidelberg, Germany; 4 Faculty of Biosciences, Heidelberg University, Im Neuenheimer Feld 234, 69120 Heidelberg, Germany; 5 Department of Medical Oncology, National Center for Tumor Diseases (NCT) and Heidelberg University Hospital, Im Neuenheimer Feld 460, 69120 Heidelberg, Germany; 6 Center for Biomedical Research and Education (ZBAF), Institute of Virology and Microbiology, Faculty of Health, School of Medicine, Witten/Herdecke University, Stockumer Straße 10, 58453 Witten, Germany; 7 Cancer Therapeutics Program, Ottawa Hospital Research Institute, 501 Smyth Road, Ottawa, ON K1H 8L6, Canada

**Keywords:** measles virus, microRNAs, miR-122, oncolytic viruses, virotherapy, Drosha, miRNA biogenesis

## Abstract

Virotherapy is a promising, novel form of cancer immunotherapy currently being investigated in pre-clinical and clinical settings. While generally well-tolerated, the anti-tumor potency of oncolytic virus-based monotherapies needs to be improved further. One of the major factors limiting the replication efficiency of oncolytic viruses are the antiviral defense pathways activated by tumor cells. In this study, we have designed and validated a universal expression cassette for artificial microRNAs that can now be adapted to suppress genes of interest, including potential resistance factors. Transcripts are encoded as a primary microRNA for processing via the predominantly nuclear RNase III Drosha. We have engineered an oncolytic measles virus encoding this universal expression cassette for artificial microRNAs. Virally encoded microRNA was expressed in the range of endogenous microRNA transcripts and successfully mediated target protein suppression. However, absolute expression levels of mature microRNAs were limited when delivered by an oncolytic measles virus. We demonstrate that measles virus, in contrast to other cytosolic viruses, does not induce translocation of Drosha from the nucleus into the cytoplasm, potentially resulting in a limited processing efficiency of virus-derived, cytosolically delivered artificial microRNAs. To our knowledge, this is the first report demonstrating functional expression of microRNA from oncolytic measles viruses potentially enabling future targeted knockdown, for instance of antiviral factors specifically in tumor cells.

## 1. Introduction

Over the past years, many different oncolytic virus (OV) platforms have emerged and oncolytic virotherapeutics have been tested in preclinical and clinical trials [1,2,3]. Despite promising results, OVs with enhanced safety and efficacy profiles are required to enable and justify a broader clinical application. Engineering strategies therefore aim at improving virus delivery, tumor cell selectivity, and oncolytic efficacy [4]. In terms of tumor cell selectivity, enhanced oncotropism can be achieved by genetic fusion of viral attachment proteins with targeting moieties such as (single chain) antibodies, or ligands for cytokine or growth factor receptors [5,6,7]. Another targeting strategy is the integration of microRNA (miRNA) target sequences into the viral genome as some tissue-specific miRNAs are downregulated during malignant transformation. Thereby, viral replication can be restricted to tumor cells. OVs specifically infect and replicate in tumor cells, leading to cell lysis when progeny particles are released from infected cells. Tumor cell killing depends on a variety of factors including direct virus-mediated toxicity and immune-mediated responses. The insertion of therapeutic transgenes such as cytokines or prodrug convertases into the viral genome (“arming”) can potentiate the oncolytic activity of OVs [5,6,7,8,9]. In addition, the combination of OVs with other treatment modalities, such as chemotherapy, radiotherapy, or checkpoint inhibition, can further enhance anti-tumor efficacy [5,9,10,11]. For several cytoplasmic RNA viruses, viral delivery of therapeutic miRNAs has been achieved, while no reports of miRNA-encoding measles viruses (MeV) have been published [12,13,14,15].

Measles vaccine strains are highly suitable for use as OVs as they have an outstanding safety profile and an inherent oncolytic potential [16]. Genetic engineering enables (re-)targeting and arming of MeV to further enhance tumor cell specificity and oncolytic efficacy [9,11,17]. In this study, MeV-mediated delivery of artificial miRNAs was envisioned as a new strategy to further enhance the oncolytic efficacy of MeV. miRNAs are small RNA molecules that can suppress selected target proteins. Viral miRNA delivery can serve to downregulate transcripts of interest which mediate anti-viral or tumorigenic effects [18,19].

The first step of canonical miRNA processing takes place in the nucleus and comprises the cleavage of primary miRNA (pri-miRNA) to precursor miRNA (pre-miRNA) by the microprocessor complex, formed by the RNase III enzyme Drosha and the DGCR8 (DiGeorge critical region 8) protein. Pre-miRNA is exported to the cytoplasm where it is cleaved by another RNase III enzyme, Dicer, which generates a miRNA duplex consisting of approximately 22 base pairs (=mature miRNA). After Dicer cleavage, the guide strand is incorporated into the RNA-induced silencing complex (RISC) thereby directing it to the target mRNA. Upon binding of RISC to the target mRNA, silencing is exerted through mRNA cleavage, translational repression, or mRNA deadenylation [20].

Several viruses, mainly double-stranded DNA viruses, have been shown to naturally encode miRNAs [21]. It was hypothesized that the majority of RNA viruses do not encode miRNAs as viral miRNA expression could result in genomic cleavage or self-targeting [22,23]. Furthermore, it was assumed that viral delivery of miRNA is limited to viruses that replicate in the nucleus, as the first step of miRNA processing takes place in the nucleus, while replication of most RNA viruses is restricted to the cytoplasm [15]. Several studies disproved this assumption and demonstrated that cytoplasmic RNA viruses such as sindbis virus (SINV) and vesicular stomatitis virus (VSV) can generate functional artificial miRNA [12,14,15]. However, it remained unclear how pri-miRNA derived from cytoplasmic viruses is processed. Shapiro et al. demonstrated that the processing of respective pri-miRNAs depends on both Drosha and Dicer cleavage and that it is exclusively localized in the cytoplasm during viral infection and miRNA processing. Furthermore, the authors could show that some viruses induce translocation of Drosha to the cytoplasm, thereby presumably enabling cytoplasmic processing of pri-miRNAs [24].

In this study, we aimed at generating miRNA encoding oncolytic MeVs (oMeVs) to provide proof of concept that oMeVs can be employed to deliver functional artificial miRNA. Regarding the biogenesis of virus-derived miRNAs, we investigated the subcellular localization of Drosha during MeV infection.

## 2. Materials and Methods

Cell Culture. Vero (African green monkey kidney) and HeLa (Human cervix adenocarcinoma) cells, obtained from ATCC, Manassas, VA, were cultured in DMEM (Life Technologies, Darmstadt, Germany) supplemented with 10% fetal calf serum (DMEM^+^) at 37 °C in a humidified atmosphere with 5% CO_2_ content. Cells were routinely tested for mycoplasma contamination.

Transfection of miRNA, siRNA, DNA, and poly(I:C). For transfection, 3.5 × 10^5^ or 1.75 × 10^4^ cells were seeded in 6- or 96-well plates, respectively. The next day, cells were transfected using the transfection reagent Lipofectamine2000 (Life Technologies) according to the manufacturer’s instructions. miRNA, siRNA, poly(I:C), and DNA were transfected at a concentration of 20 nM, 20 nM, 10 µg/mL, and 0.5 µg/mL, respectively.

Recombinant MeVs. Genetically engineered, double-stranded DNA fragments encoding the miRNA expression cassette MeVami-122 were cloned into linearized pCG plasmid (eukaryotic expression vector) and pcpNSe plasmids (antigenomic plasmids derived from the Measles virus Edmonston B vaccine strain). The insertion of transgenes was enabled by additional transcription units which had been integrated into the viral genome and comprise restriction sites, as well as gene start and end signals. Infectious viral particles were generated as described previously [9]. Virus stocks from the third passage were used for experiments.

Virus Titration. Serial 1:10 dilutions of virus stock solution in DMEM^+^ were performed in a 96-well plate (in octuplicates). To each well, 2 × 10^4^ Vero cells in 100 µL DMEM^+^ were added. Forty-eight h p.i. syncytia were counted, and the viral titers were calculated as cell infectious units/mL (ciu/mL).

Transgene Expression. Vero cells were seeded in 6-well plates (3.5 × 10^5^ cells/well). The next day, cells were transfected or infected with recombinant MeVs at different multiplicities of infection (MOIs). In total, 22 to 34 h post-transfection (p.t.)/p.i., cells were harvested and subjected to RNA isolation using the RNeasy Mini Kit (Qiagen, Hilden, Germany). cDNA synthesis was performed with the Maxima H Minus First Strand cDNA Synthesis Kit (Thermo Fisher Scientific, Waltham, MA, USA) using an oligo-dT primer. Subsequent PCR amplification was performed using the pri-miR-122 forward (5′-TGGTGGAATGTGGAGGTGAAG-3′) and pri-miR-122 reverse (5′-GCTCAAAGCAAACGATGCCAAG-3′) primers which bind within the miRNA expression cassette. The size of the PCR products was assessed by agarose gel electrophoresis.

Multi-Step Virus Growth Curve. 3 × 10^5^ Vero cells per well were seeded onto 6-well plates. The next day, cells were infected at an MOI of 0.03 with either MeV-ld-EGFP, MeV-ld-MeVami-122 H-EGFP, MeV-ld-EGFP H-MeVami-122, or MeV-ld-EGFP P-MeVami-122. Cells were harvested in the medium at 24, 36, 48, 72, and 96 h p.i. After one freeze-thaw cycle, the progeny virus was quantified by titration, as described above. The mean values of two independent experiments are plotted and error bars indicate standard deviation.

Absolute quantification of miRNA. Vero cells were seeded in 6-well plates (3.5 × 10^5^ cells/well). The next day, cells were transfected with pCG MeVami-122, control agent miR-122, or infected with recombinant MeVs at an MOI of 0.3. Thirty h p.i./p.t. cells were harvested. Isolation of total RNA was performed using the miRNeasy Mini Kit (Qiagen, Hilden, Germany) according to the manufacturer’s instructions. For the generation of the miRNA standard curve, miRNA with the respective sequence was obtained as 5′ phosphorylated RNA oligonucleotides. By serial dilution, standards containing 10^3^ to 10^10^ miRNA molecules were prepared. Standards were complemented with *E. coli* total RNA (Sigma-Aldrich, St. Louis, MO, USA) at an equal amount as the sample RNA used for reverse transcription. Prior to the reverse transcription reaction, miRNAs were polyadenylated using the *E. coli* Poly(A) polymerase (New England Biolabs, Frankfurt, Germany). Subsequent cDNA synthesis was performed using the miScript II RT Kit (Qiagen). qPCR was performed in technical triplicates on a LightCycler 480 (Roche, Mannheim, Germany) using the miScript SYBR Green PCR Kit (Qiagen) and a miR-122 -specific primer (5′-UGGAGUGUGACAAUGGUGUUUG-3’). Normalization controls were conducted for each sample using the miScript PCR Control (RNU6-2, Qiagen) primer. Crossing point (CP) values were calculated by the LightCycler software (software version LCS480 1.5.0.39, Roche) using the second derivative maximum method. A standard curve was generated from CP values and the respective miRNA input. Linear regression was performed for the linear range of the curve. The respective linear equation was used to calculate the number of miRNA molecules in the qPCR reaction. Before, the CP values of samples were normalized using RNU6-2 normalization controls. Knowing the number of cells used for RNA purification, their total RNA amount as well as the RNA input into the reverse transcription reaction, the number of cells used for cDNA synthesis and subsequent qPCR was calculated. By dividing the number of calculated miR-122 molecules used in the qPCR reaction by the number of analysed cells, the exact number of miR-122 molecules per cell could be determined. Mean values are plotted and depicted with the standard deviation.

Relative quantification of pri-miRNA and miRNA. Pri-miRNA was isolated from 3.5 × 10^5^ Vero, HEK293 or PC9 cells treated as described above using the RNeasy Mini Kit (Qiagen). DNase digestion was performed on-column prior to RNA cleanup. Reverse transcription of isolated RNA was performed using the Maxima H Minus First Strand cDNA Synthesis Kit using oligo(dT) primers (Thermo Fisher Scientific, Waltham, MA, USA). qPCR was performed on a CFX96 Touch Real-Time PCR Detection System (Bio-Rad, München, Germany) using the *Power*SYBR Green PCR Master Mix (Life Technologies, Warrington, UK) and pri-miR-122-specific primers (pri-miR-122 for 5′-TGGTGGAATGTGGAGGTGAAG-3′, pri-miR-122 rev 5′-GCTCAAAGCAAACGATGCCAAG-3′). L19 (for 5′-ACCCCAATGAGACCAATGAAT-3′ and rev 5′-CAGCCCATCTTTGATGAGCTT-3′) was used as an endogenous housekeeping gene. Mature miRNA was isolated and processed as described above using the Qiagen miRNeasy Kit for RNA isolation followed by the miScript II PCR system for reverse transcription and qPCR detection. For both primary and mature miRNA, qPCR was performed in biological replicates. Relative pri-miRNA and miRNA expression was determined using the 2^−ΔΔ*CP*^ method, normalizing to pCG-delivered miRNA [25]. Values thus represent relative expression over pCG MeVami-122 transfected samples.

Luciferase Assay. Vero cells (1.75 × 10^5^ cells/well, 96-well plate) were transfected with a psiCHECK™-2-miRTS-122 vector encoding the two reporter enzymes firefly Luciferase (control reporter) and Renilla Luciferase (experimental reporter), the latter harboring a miR-122 target site (miRTS-122) within its 3′ UTR. Cells were either co-transfected with pCG MeVami-122/a control (miR-122) or infected at MOI 0.3 with recombinant MeVs carrying the MeVami-122 cassette/a control virus (MeV ld-EGFP) (6 h p.t.). Read out was performed 30 h p.i. on a Tecan microplate reader (Infinite M200 Pro microplate reader with i-control 1.6 Software, Tecan, Männedorf, Switzerland) using the Dual-Glo Stop and Glo Luciferase Assay System (Promega, Mannheim, Germany). Cells exclusively transfected with the psiCHECK™-2-miRTS-122 vector acted as a negative control (maximal Renilla luminescence) while cells transfected with miR-122 were used as positive control (minimal Renilla luminescence). Relative response ratios (RRR) were determined using the following formula:

(1) $$RRR=\frac{\left(experimental\;sample\;ratio\right)-positive\;control\;ratio}{negative\;control\;ratio-positive\;control\;ratio}$$

Immunofluorescence staining. For immunofluorescence staining, cells were seeded onto glass coverslips in 6-well plates. The next day, cells were transfected with poly(I:C) or infected with MeV ld-EGFP at an MOI of 0.3. After 6 h, the medium was renewed and supplemented with fusion-inhibitory peptide (FIP, N-CBZ-D-PHE-PHE-GLY, Sigma-Aldrich) for infected cells. Twenty-four h later, cells were washed with DPBS and fixed with 4% paraformaldehyde in DPBS for 10 min. Cells were washed with DPBS and permeabilized with 0.2% Triton X-100 in DPBS for 15 min. For blocking, cells were treated with 2% BSA, and 0.1% Triton X-100 in DPBS for 1 h. Primary antibody (rabbit anti-Drosha, Abcam ab12286; 1:250) was added and incubated overnight at 4 °C. Cells were washed with DPBS followed by incubation with secondary antibody (goat anti-rabbit AlexaFluor594, Thermo Fisher Scientific, Waltham, MA, USA, A11012; 1:500) and DAPI for 1.5 h. Cells were washed with DPBS and mounted onto microscope slides with mounting medium (S3023, Agilent, Santa Clara, CA, USA). After hardening, slides were imaged on a Zeiss Cell Observer (Carl Zeiss, Jena, Germany).

## 3. Results

### 3.1. Genetic Design, Transgene Expression, and Replication Kinetics of miR-122-Encoding MeV

**Figure 1:**
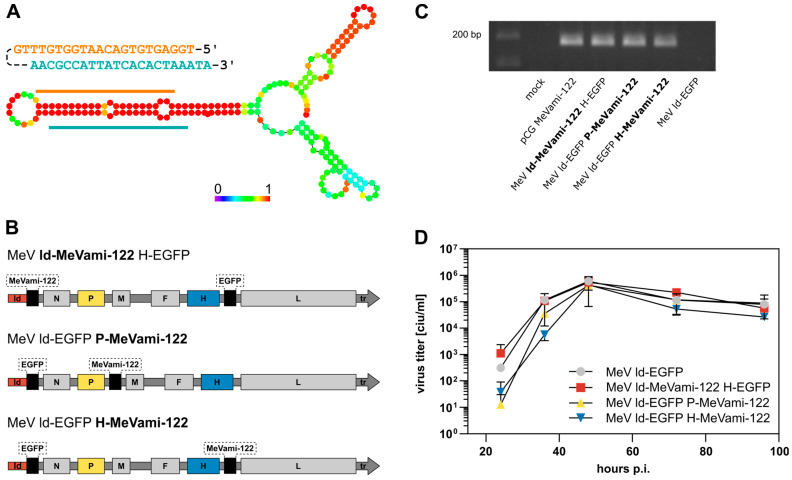
Design of MicroRNA-encoding MeV. (**A**) Depiction of MeVami-122 expression cassette. The pre-miR-122 sequence comprises the miR-122 sequence (orange) and its complementary target site sequence (blue). The precursor sequence was elongated by 50 nucleotides 5′, and 49 nucleotides 3′ of pri-miR-122, ensuring both the recognition by the microprocessor and the polyhexameric length of the viral genome upon integration of the cassette. Below: Secondary structure of the MeVami-122 cassette, as predicted by the “RNAfold webserver” of the University of Vienna [29]. Colors indicate base pairing probabilities, wherein red indicates a base pairing probability of 100%. (**B**) Schematic depiction of the three MeV genomes harboring the MeVami-122 cassette. EGFP and MeVami-122 transgenes were inserted at the indicated positions. (**C**) Transgene expression of MeVami-122-encoding MeV. Vero cells were either transfected with pCG MeVami-122 or infected with one of the MeVami-122 encoding viruses. Samples from RT-PCR performed with pri-miR-122 specific primers were run on an agarose gel. (**D**) Virus growth curves. Vero cells were infected with the indicated recombinant MeV variant (MOI 0.03) and titers of progeny virus were determined at the indicated time points. The mean of *n* = 2 experiments is plotted. Error bars show standard deviation.

To enable the expression of artificial miRNA by oMeV, we designed an miRNA expression cassette (MeVami-122; see Supplementary Figure S1). Analogous to previous studies employing cytoplasmic RNA viruses for the expression of artificial miRNA, miR-122 was selected for establishing the expression cassette (Shapiro et al., 2012) [24]. The design of MeVami-122 is based on the miR-122 backbone consisting of the precursor miR-122 sequence flanked by 50 and 49 nucleotides of primary miR-122 (pri-miR-122) at its 5′ and 3′ ends, respectively. Furthermore, as required for genome replication in *Paramyxoviridae*, we ensured a polyhexameric length of the viral genome upon cassette integration (“rule of six”) [26]. In the process of miRNA biogenesis, the miRNA duplex strand which is thermodynamically less stable at its 5′ ends is preferably loaded into the RISC, thus becoming the active guide strand [27]. Considering this, we designed the MeVami-122 cassette in accordance:. Figure 1A depicts the secondary structure as predicted by the “RNAfold webserver” provided by the University of Vienna [28,29]. Base pair probabilities indicate that the strand with the miR-122 sequence has the highest probability to becoming the guide strand [28].

As depicted in Figure 1B, the expression cassette was inserted into one of three additional transcription units (ATUs) within the viral genome, namely upstream of the MeV *N* gene, downstream of the MeV *P* gene, or downstream of the MeV *H* gene, respectively. All viruses were successfully cloned, rescued, and propagated to obtain high-titer stock solutions. A MeVami-122-encoding expression plasmid (pCG MeVami-122) was constructed as a control for further experiments. Transgene expression was confirmed on the RNA level for all recombinant viruses (Figure 1C).

One-step-growth curves were generated to assess the potential impact of the inserted miRNA expression cassette on viral replication kinetics. All MeVami-122-harboring viruses showed similar peak titers and replication dynamics when compared to the parental virus stain (MeV ld-EGFP) suggesting that the insertion of MeVami-122 does not substantially affect replication kinetics in Vero cells (Figure 1D).

### 3.2. Cells Infected with miRNA-Encoding MeV Produce Functional miRNAs, Albeit at Comparatively Low Copy Numbers

**Figure 2:**
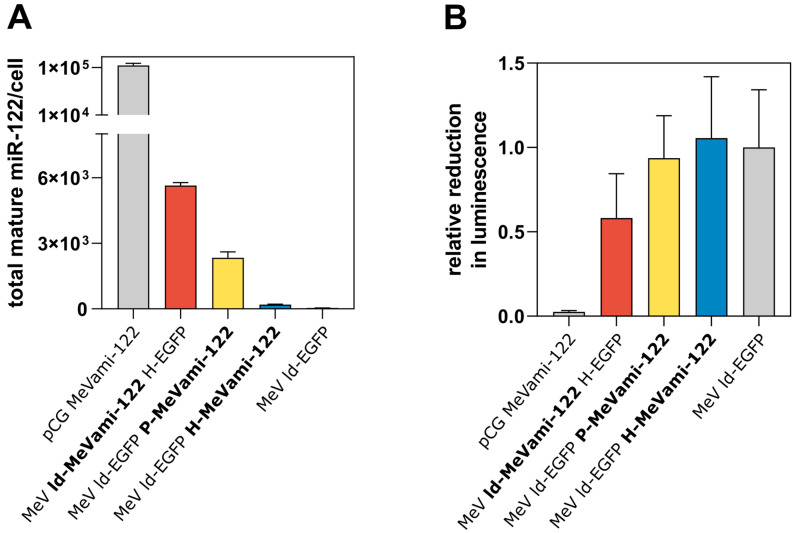
Expression of MiRNA and functional target protein suppression. (**A**) qPCR-based absolute quantification of miR-122 in cells transfected or infected with MeVami-122-encoding vectors. Vero cells were either transfected with 0.5 μg/mL pCG MeVami-122 or infected with MeVami-122-encoding viruses at MOI 0.3 and harvested 30 h p.t./p.i. qPCR was carried out in technical triplicates. Mean values are plotted. Error bars indicate standard deviation. (**B**) Target protein suppression in cells transfected or infected with MeVami-122-encoding vectors. Vero cells were transfected with psiCHECK™-2-miRTS-122 and either co-transfected with pCG MeVami-122 or infected with MeVami-122-encoding viruses at MOI 0.3 (6 h p.t.). Thirty h p.t./p.i. Renilla- and firefly luciferase-mediated luminescence was measured. Relative response ratios (RRRs) were calculated using negative (mock) and positive (miR-122) controls. Mean values of biological replicates (*n* = 3) are plotted. Error bars indicate standard deviation. Values represent RRRs relative to the RRR of a control virus (MeV ld-EGFP).

Having verified that pri-miR-122 encoded by MeV is expressed and does not substantially affect viral replication kinetics, we next wanted to assess the expression of mature miR-122 via MeVami-122-harboring viruses. To this end, a previously established qPCR-based method for the absolute quantification of miRNA was applied [30]. Vero cells were infected with recombinant viruses (or transfected with a miRNA-encoding plasmid) and the copy number of mature miR-122 was determined by qPCR 30 h post-infection (p.i.), when cells were completely in syncytia indicating widespread infection. Although viral titers are higher after infection (see Figure 1D), intact host cell machinery is required to assess the effect of virus-derived miRNA. Thus, for all subsequent functional analysis, cells were analyzed at this time point. Compared to the copy number of mature miR-122 generated after transfection of the miRNA-encoding control plasmid pCG-MeVami-122 (103,901 molecules/cell), the copy number of miRNA generated by miRNA-encoding viruses was relatively low (Figure 2A). MeV ld-MeVami-122 H-EGFP produced the greatest copy number of mature miR-122 (5645 molecules/cell) among the miRNA-encoding viruses. Gel electrophoresis with subsequent sequencing confirmed that qPCR products corresponded to mature miR-122 (data not shown).

To investigate whether the comparatively small copy number of virus-derived (mature) miR-122 molecules is functional, target protein suppression was assessed through a luciferase-based reporter assay system. The assay vector psiCHECK™-2 employs two different reporter enzymes: Renilla and firefly luciferase. The former can be regulated by an inserted miRNA target site (miRTS). For the construction of psiCHECK™-2-miRTS-122, a miRTS harboring three perfectly complementary target sequences for miR-122 was cloned into the assay vector (Figure S2). Vero cells were transfected with psiCHECK™-2-miRTS-122 and either co-transfected with the miR-122-encoding plasmid (pCG MeVami-122) or subsequently infected with MeVami-122-encoding viruses. As shown in Figure 2B, MeV ld-MeVami-122 H-EGFP successfully mediates target protein suppression, observable by a reduction of luciferase activity by ~40%, compared to the control virus condition. The other two recombinant viruses, which produce less mature miRNA, do not suppress luciferase activity. Of note, luciferase activity was almost fully suppressed in cells transfected with the miRNA-encoding plasmid pCG MeVami-122 (which produces significantly more mature miR-122) suggesting that the extent of target protein suppression correlates with the copy number of produced mature miRNA. Collectively, these results indicate that recombinant MeV can deliver mature, functional miRNAs, albeit at comparatively low copy numbers.

### 3.3. Limited Processing of MeV-Encoded miRNA

**Figure 3:**
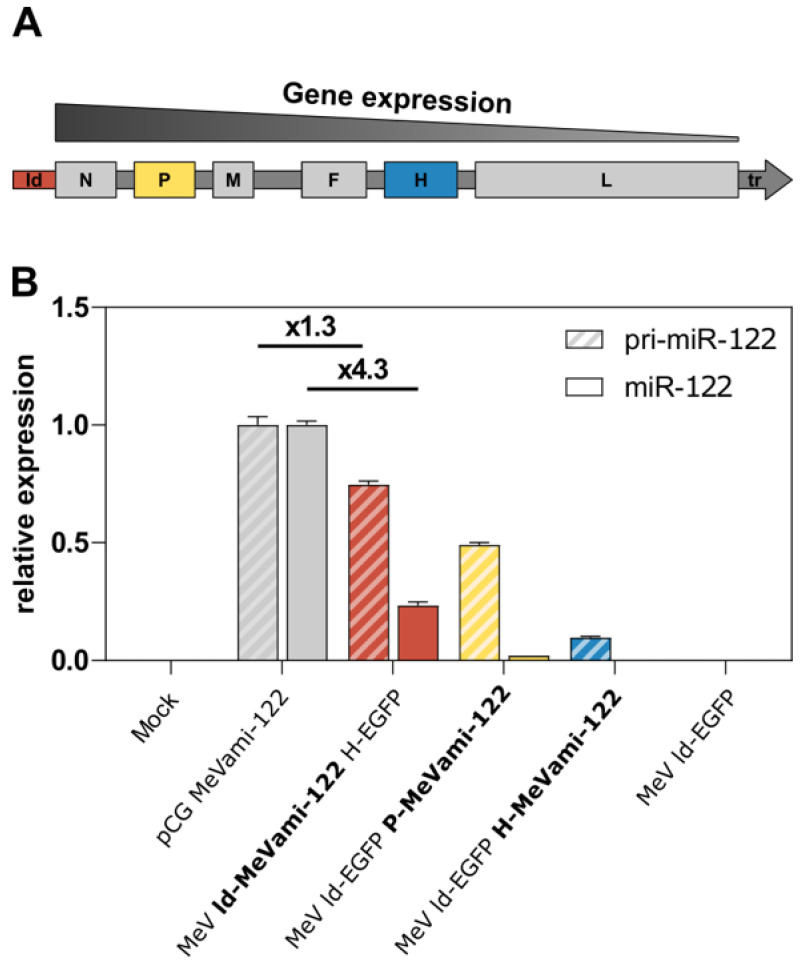
Transcriptional gradient and differential expression of (pri-)miR-122. (**A**) Schematic depiction of MeV genome with indicated gene expression gradient. Genes further upstream in the genome are transcribed more frequently. (**B**) Relative expression of pri-miR-122 and mature miR-122 in cells transfected or infected with MeVami-122-encoding vectors. Vero cells were either infected with recombinant, MeVami-122-encoding MeV at an MOI of 0.3 or transfected with 0.5 μg/mL pCG MeVami-122 and harvested 30 h p.i./p.t. Results of qPCR analysis are depicted as viral pri-miR-122/mature miR-122 expression relative to pri-miR-122/mature miR-122 expression, respectively, in transfected cells (pCG MeVami-122).

Considering the previous findings, we subsequently investigated why the copy number of mature miRNA produced upon infection with miRNA-encoding MeV was limited, especially when compared to plasmid transfection. As confirmed by qPCR (Figure 2A), the copy number of MeV-derived miRNA correlates with the genomic position of the expression cassette which is a known feature of paramyxovirus biology often referred to as the “transcriptional gradient” [31] (Figure 3A). While the transcriptional gradient explains the differences in virus-derived miRNA expression, we wanted to assess whether the large discrepancy in mature miR-122 expression levels between the plasmid expression vector and recombinant viruses was due to a low viral transcription efficiency of the expression cassette. To this end, qPCR-based relative quantification of the pri-miR-122 transcript was performed. Interestingly, the difference in expression of pri-miR-122 between recombinant viruses and plasmid expression vectors was considerably smaller when compared to the largely diverging expression levels of mature miRNA (Figure 3B). While pCG MeVami-122 generates only 1.3 times more pri-miR-122 than MeV ld-MeVami-122 H-EGFP, it generates 4.3 times more mature miR-122. This discrepancy suggests that impaired processing rather than reduced transcription efficiency is the cause of low expression of mature miRNA in the context of recombinant MeV.

### 3.4. Nuclear Localization of Drosha in MeV-Infected Cells Might Be Responsible for the Limited Processing of MeV-Derived miRNA

**Figure 4:**
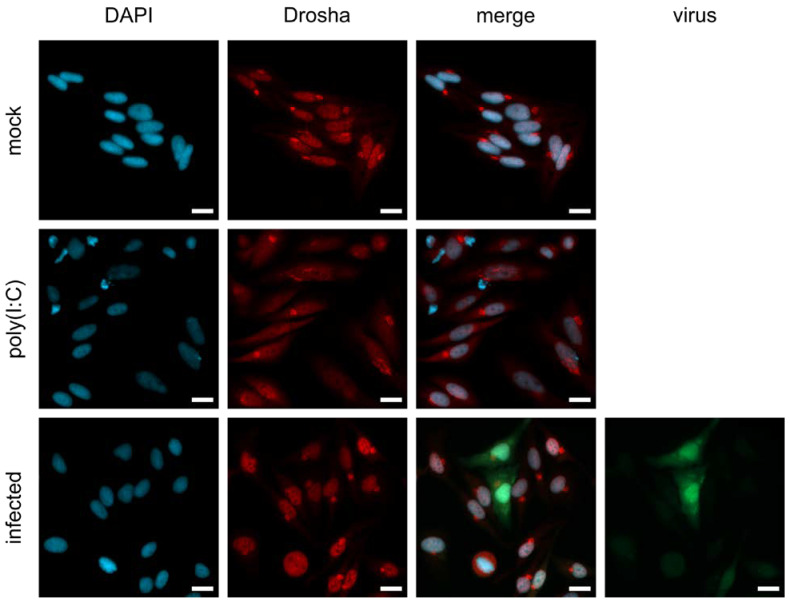
Localization of Drosha upon MeV infection. Nuclear localization experiments with immunofluorescence staining of Drosha. HeLa cells were mock-treated, and treated with 10 μg/mL poly(I:C) or infected with MeV ld-EGFP at MOI 0.3 (green). Drosha (red) and the nucleus (blue) were stained with antibody and DAPI, respectively. Bright spots in the Drosha staining are considered staining artifacts. Scale bar: 20 µm.

Previous results from other groups have demonstrated that cytoplasmic processing of miRNA derived from other cytosolic RNA viruses such as VSV and SINV is enabled by the translocation of Drosha from the nucleus to the cytoplasm [24,32]. To assess Drosha localization during infection with MeV, we performed immunofluorescence (IF) staining experiments. We used interferon-competent HeLa cells since dsRNA recognition was discussed as a prerequisite for Drosha translocation, although it was shown that the relocalization of Drosha is independent of IFN-I signaling [32]. Cells were infected with MeV and fusion-inhibitory peptide (FIP) was added to inhibit syncytia formation, a technical prerequisite for IF staining. The synthetic dsRNA analogue poly(I:C) was used as a positive control as it has been shown to induce Drosha translocation from the nucleus to the cytoplasm [32]. Infected, as well as poly(I:C)-treated, HeLa or Vero control cells were stained for nuclei, Drosha, and virus-derived EGFP (Figure 4 and Figure S3). While Drosha was located in the cytoplasm of poly(I:C)-treated cells, we could not detect Drosha in the cytoplasm of MeV-infected cells, suggesting that MeV, in contrast to certain other RNA viruses, does not induce the translocation of Drosha to the cytoplasm. As Drosha translocation has previously been identified as a prerequisite for cytoplasmic processing of RNA virus-derived miRNA [24], the unchanged nuclear localization of Drosha upon MeV infection could explain the observed impaired processing of virus-derived artificial miRNA.

Given the evidence that poly(I:C) induces Drosha translocation to the cytoplasm, we sought to determine whether treatment with poly(I:C) resulted in higher concentrations of mature miRNA in cells infected with recombinant MeV. However, we saw drastically decreased viral infection and increased cytotoxicity of infected HeLa cells, when pretreated with poly(I:C). This is likely due to the induction of anti-viral signaling by poly(I:C). Therefore, this approach could not be used in the respective context. To investigate whether the cytoplasmic presence of Drosha increases the copy number of MeV-derived miRNA, we employed cell lines with differential Drosha localization [33]. As described by Link et al., PC9 cells show cytoplasmic localization of Drosha even in non-induced states, while HEK293 cells express Drosha almost exclusively in the nucleus [33]. In our hands, PC9 cells showed a tendency towards increased cytoplasmic Drosha localization, compared to HEK293 (Figure S4). However, the difference was not striking. We nevertheless tested whether this effect was sufficient to increase the processing efficiency of virus-delivered miRNA in PC9 cells when compared to HEK293 and Vero cells. In the end, miRNA biogenesis was equally efficient in all three cell lines (Figure S5). Fold change of primary to mature miRNA varied between 5.7 and 7.8, with PC9 cells showing an intermediate fold change of 6.6. We were thus not able to confirm our hypothesis of improved processing of MeV-encoded miRNA in PC9 cells.

## 4. Discussion

This is the first study describing the generation of an engineered oMeV encoding functional, artificial miRNA. Various applications can be envisioned, including the delivery of miRNAs targeting mediators of antiviral signaling to increase viral replication.

miRNA expression levels from recombinant viruses correlated with the genomic insertion site of the miRNA expression cassette according to the MeV transcriptional gradient. The extent of target protein suppression in a luciferase-based assay system correlated with miRNA expression levels, since only the recombinant virus expressing the greatest amount of miRNA reduced the target protein activity considerably. Notably, cells transfected with an expression plasmid generating abundant copy numbers of mature miRNAs showed almost complete target protein suppression.

Assuming a concentration-dependent effect, the number of miRNA molecules required for sufficient target protein suppression likely depends on the target protein. In the luciferase-based reporter assay, a clear knockdown of the target protein could only be detected in case of infection with MeV harboring the miRNA cassette in the most upstream ATU (MeV ld-MeVami-122 H-EGFP). Presumably, the luciferase-based assay system was not sensitive enough to detect the silencing effect of lower copy numbers of mature miRNA as expressed by the other two viruses (MeV ld-EGFP P-MeVami-122, MeV ld-EGFP H-MeVami-122). Considering that the reporter luciferase gene harbors a target sequence which contains three perfectly complementary miRNA target sequences and is constitutively expressed under a strong promoter, its expression is very high and may only be suppressed by an abundant copy number of miRNA—as generated by the miRNA expression plasmid. While lower copy numbers of mature miRNAs (as expressed via MeV ld-EGFP P-MeVami-122 and MeV ld-EGFP H-MeVami-122) were not sufficient to induce target protein suppression in this artificial reporter assay, we hypothesize that expression of viral miRNAs at levels comparable to endogenous miRNA expression—which is the case for all three recombinant viruses—could nevertheless result in meaningful target protein suppression in a more physiological setting [34]. However, other oncolytic RNA viruses were shown to generate considerably higher amounts of mature miRNA when compared to MeV. For example, expression levels of miRNA delivered by SINV and VSV were estimated to range from 28,000 to 55,000 miRNA copies/cell [12,24]. In this regard, it is noteworthy that these viruses were shown to induce the translocation of Drosha from the nucleus to the cytoplasm which has been identified as one underlying mechanism of cytoplasmic processing of virus-derived miRNA [13,24,32]. In contrast, our study indicates that Drosha does not translocate from the nucleus to the cytoplasm upon MeV infection. Therefore, we hypothesize that the lack of a cytoplasmic microprocessor complex could potentially contribute to the comparatively low expression levels of MeV-derived mature miRNA.

While the exact mechanism of Drosha translocation remains unclear, we confirmed that poly(I:C), which is a synthetic analogue of dsRNA (a common viral pathogen-associated molecular pattern), induces the translocation of Drosha to the cytoplasm. dsRNA is generated during the viral replication cycle of most viruses and plays a key role in the TLR3-mediated activation of antiviral innate immunity [35]. Of interest, it was assumed that in contrast to DNA and positive-strand-RNA viruses, negative-strand viruses either do not generate detectable levels of dsRNA during their replication process or employ mechanisms to mask accumulating dsRNA [35]. However, Son et al. detected dsRNA in cells infected with negative-strand RNA viruses, including VSV and MeV, albeit to a much lesser extent when compared to positive-strand RNA viruses [35]. Remarkably, infection with recombinant MeV lacking the C protein results in the accumulation of viral dsRNA which was shown to trigger the antiviral immune response by activation of the dsRNA-dependent protein kinase R, causing impaired viral growth [36]. This indicates that the MeV C protein impedes the production of dsRNA to circumvent the antiviral immune response which may contribute to the lacking translocation of Drosha to the cytoplasm upon MeV infection. Accordingly, VSV is an exception to other negative-strand RNA viruses as it accumulates excessive amounts of dsRNA during its replication cycle which triggers the host cell immune response and has been shown to induce the translocation of Drosha to the cytoplasm, thus allowing the generation of high copy numbers of virus-derived miRNA [13,37].

Limiting Drosha translocation to the cytoplasm might be more than a mere byproduct of evading TLR3-mediated antiviral effects, and could be a mechanism actively employed by MeV. We showed that the processing of virus-delivered miRNA is not improved in a cell line which is known for cytosolic localization of Drosha in untreated cells [33]. Of note, RNA interference (RNAi) is the major response to virus infection in plants, nematodes, and arthropods, whereas, in vertebrates, IFN signaling is the predominant antiviral response strategy [32]. However, evidence for antiviral RNAi in mammals was reported by Maillard et al., whereby it was the coexistence of these two antiviral mechanisms was suggested [38]. Nuclear localization of Drosha requires phosphorylation at two sites, namely at Serine300 and Serine302 [39,40]. Inversely, its translocation to the cytoplasm requires dephosphorylation at these sites [32]. It might be assumed that, at least for some RNA viruses including VSV and SINV, the translocation of Drosha to the cytoplasm is facilitated by a virus-induced phosphatase that has yet to be identified. It is conceivable that MeV interferes in this balance of phosphorylation and dephosphorylation towards a nuclear localization of Drosha to circumvent antiviral RNAi. In-depth analysis of this signaling axis could elucidate a novel immunosuppressive mechanism of MeV and present targets to improve processing of MeV-derived miRNAs.

However, although limited, miRNA biogenesis in the context of MeV does occur, potentially through non-canonical pathways which comprise Drosha- as well as Drosha- and Dicer-independent miRNA processing [41,42]. Alternatively, it is imaginable that pri-miRNA translocates to the nucleus where it gains access to the microprocessor or that only very low levels of Drosha do translocate to the cytosol upon MeV infection. Further studies are required to gain more insights into the processing of MeV-derived miRNA and to unravel the role of Drosha in the response to virus infection. In summary, this study describes for the first time the generation of oMeV encoding functional, artificial miRNA, and thus presents a new tool in the oMeV toolbox. Going forward, the miRNA expression cassette for MeV established herein can be further engineered with the goal of knocking down host cell restriction factors to potentially increase the efficacy of oMeV therapy. Promising targets of artificial miRNAs thus comprise RIG-I andMDA-5 as well as other signaling molecules of the IFN pathway mediating antiviral signaling [43,44]. Furthermore, virus-mediated RNAi could downregulate cancer-related mRNAs in tumor cells. In this context, the expression of miR-34, which negatively regulates the anti-apoptotic Bcl-2 gene and is positively correlated with activity of the tumor suppressor p53, could be envisioned [18,45]. If successful, novel miRNA-encoding oMeVs might represent promising candidates for clinical translation.

## Data Availability

All data presented in this research article is available upon request to the corresponding author.

## Supplementary Materials

The following supporting information can be downloaded at https://www.mdpi.com/article/10.3390/v15020308/s1, Figure S1: MeVami-122 cassette design. All sequences are notated from 5′ to 3′. Figure S2: Schematic depiction of psiCHECK™-2-miRTS-122. The miRTS-122 target sequence consists of three perfectly complementary target sites for miR-122 that are separated by three stuffer nucleotides to increase miRNA binding. It is integrated within the 3′ UTR of the Renilla luciferase gene; Figure S3: Localization of Drosha upon MeV infection in Vero cells. Vero cells were mock-treated, treated with 10 μg/mL poly(I:C) or infected with MeV ld-EGFP (MOI = 0.3; green). Figure S4: Localization of Drosha in PC9 and HEK-293 cells upon poly(I:C) treatment or MeV-infection. HEK-293 and PC9 cells were mock-treated, treated with 10 µg/mL poly(I:C) or infected with MeV ld-EGFP (MOI = 0.3; green). Drosha (red) and the nucleus (blue) were stained with antibody and DAPI, respectively. The scale bar corresponds to 20 µm. Figure S5: PC9, HEK293 and Vero cells were either infected with MeV ld-MeVami-122 H-EGFP (MeVami-122) at MOI 0.3 or transfected with 1 µg pCG MeVami-122 and harvested 25–30 h p.i., when cells were completely in syncytia. Mature and primary miRNA was isolated and analyzed via qPCR in technical triplicates. Relative expression to pCG-derived miRNA is shown. Mean values of *n* = 2 experiments are plotted. Error bars indicate standard deviation.
