# Supplementary material for: Oncolytic Measles Virus Encoding MicroRNA for Targeted RNA Interference
Source: Viruses. 2023 Jan 22;15(2):308. doi: 10.3390/v15020308 (PMC9964028; doi:10.3390/v15020308)
Supplement: Supplementary file 1 [file viruses-15-00308-s001.zip › viruses-2166564-supplementary.pdf]

A

CCTTAGCAGAGCTG**TGGAGTGTGACAATGGTGTTTG**TGTCTAAACTATCA**AACGCCATTATCACA**
CTAAATAGCTACTGCTAGGC

B

CAATGGTGGAATGTGGAGGTGAAGTTAACACCTTCGTGGCTACAGAGTTTCCTTAGCAGAGCTG**T**
GGAGTGTGACAATGGTGTTTGTGTCTAAACTATCA**AACGCCATTATCACACTAAATA**GCTACTGC
TAGGCAATCCTTCCCTCGATAAATGTCTTGGCATCGTTTGCTTTGAGCAAGAAG

C

CAACCA*CGCGCGCG*ACCA*GGCGCGCC*CAATGGTGGAATGTGGAGGTGAAGTTAACACCTTCGTGG
CTACAGAGTTTCCTTAGCAGAGCTG**TGGAGTGTGACAATGGTGTTTG**TGTCTAAACTATCA**AACG**
CCATTATCACACTAAATAGCTACTGCTAGGCAATCCTTCCCTCGATAAATGTCTTGGCATCGTTT
GCTTTGAGCAAGAAG*GGCGCGCC*ACCA*CGCGCGCG*CAACCA

Figure S1

MeVami-122 cassette design. All sequences are notated from 5’ to 3’. (A) Depiction of the pre-miR-122 sequence comprising the miR-122 sequence (orange) and its complementary target site sequence (blue). (B) The precursor sequence was elongated by 50 nucleotides 5’ and 49 nucleotides 3’ of pri-miR-122, ensuring both the recognition by the microprocessor and the polyhexameric length of the viral genome upon integration of the cassette. (C) MeVami-122 cassette. *MauBI* and *AscI* restriction sites were integrated for cloning into antigenomic viral plasmids. Stuffer nucleotides were added 5’ and 3’ of the sequence to allow restriction enzymes to cut properly.

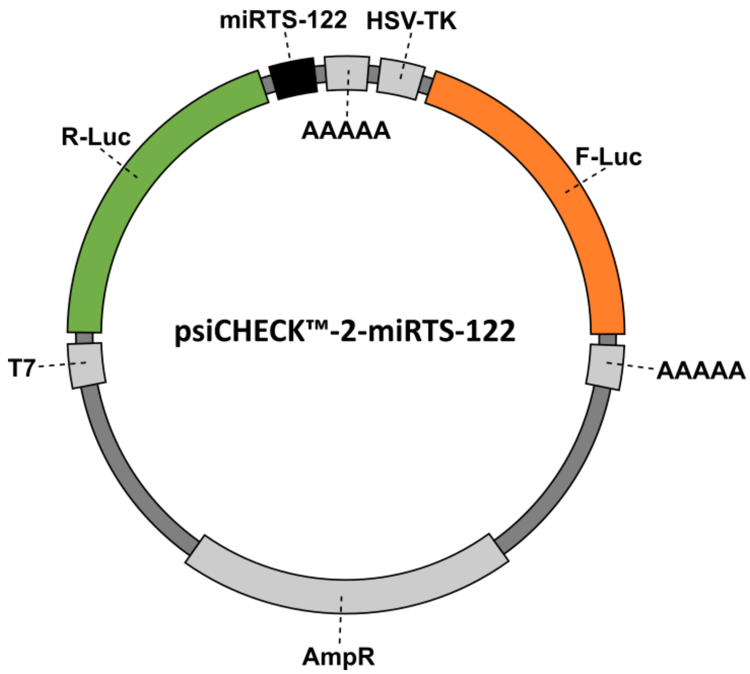

Figure S2

Schematic depiction of psiCHECK™-2-miRTS-122. The miRTS-122 target sequence consists of three perfectly complementary target sites for miR-122 that are separated by three stuffer nucleotides to increase miRNA binding. It is integrated within the 3’ UTR of the Renilla luciferase gene.

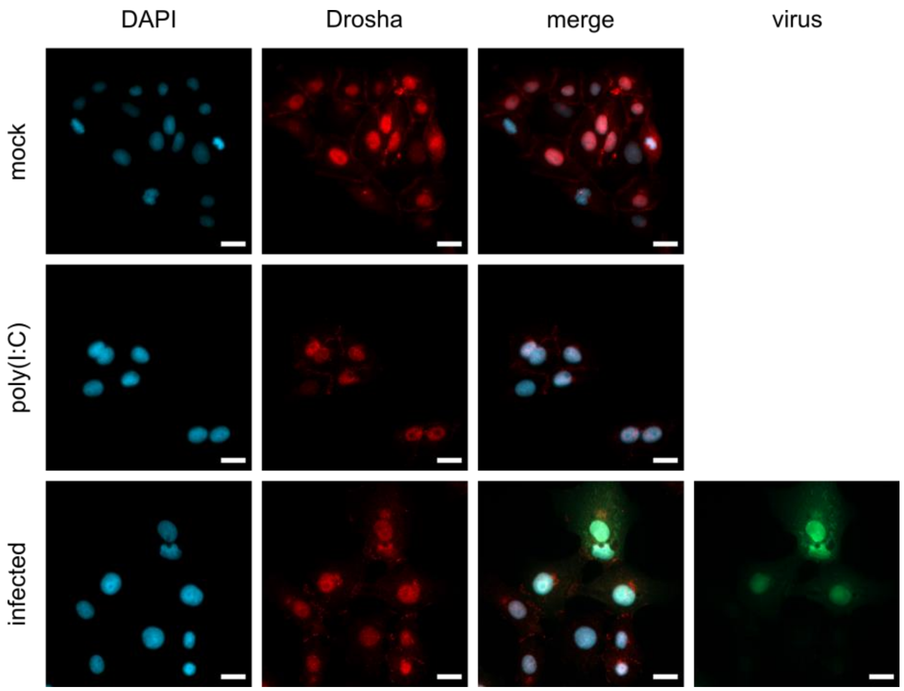

Figure S3

Localization of Drosha upon MeV infection in Vero cells. Vero cells were mock-treated, treated with 10 µg/ml poly(I:C) or infected with MeV ld-EGFP (MOI = 0.3; green). Drosha (red) and the nucleus (blue) were stained with antibody and DAPI, respectively. The scale bar corresponds to 20 µm.

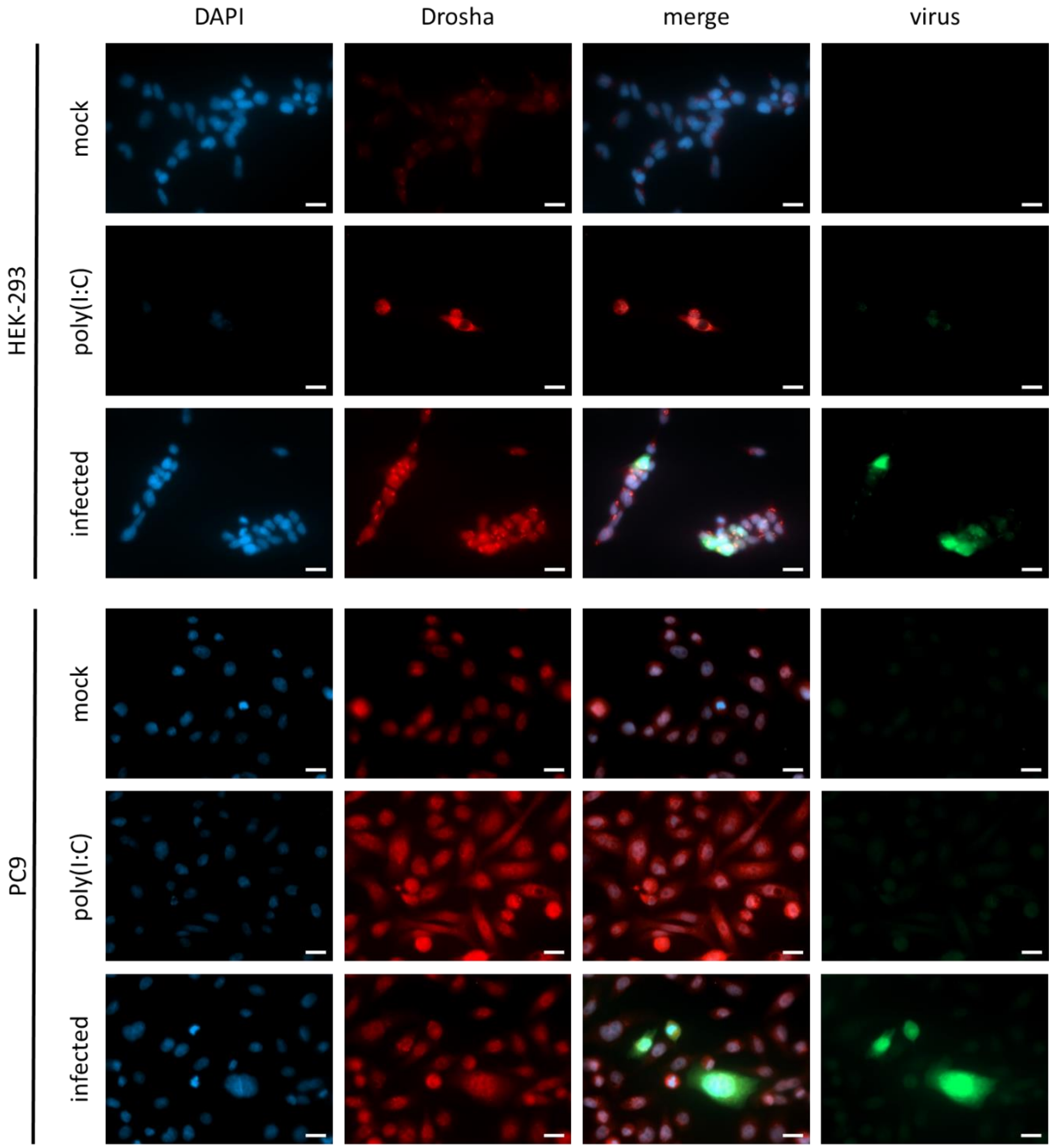

Figure S4

Localization of Drosha in PC9 and HEK-293 cells upon poly(I:C) treatment or MeV-infection. HEK-293 and PC9 cells were mock-treated, treated with 10 µg/ml poly(I:C) or infected with MeV ld-EGFP (MOI = 0.3; green). Drosha (red) and the nucleus (blue) were stained with antibody and DAPI, respectively. The scale bar corresponds to 20 µm.

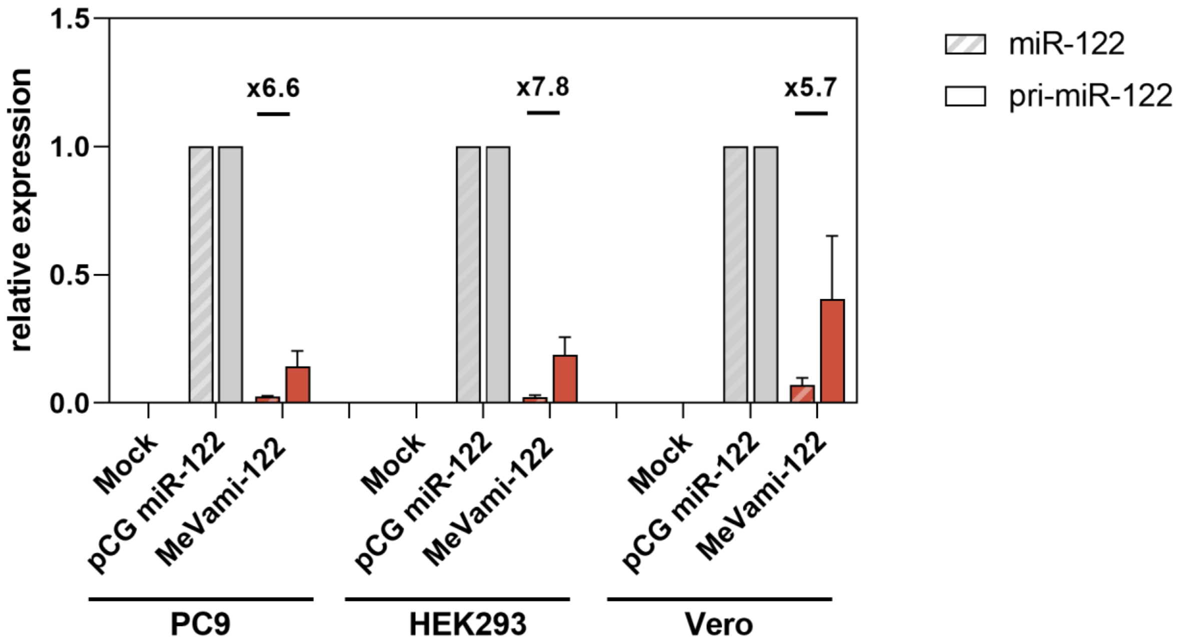

Figure S5

PC9, HEK293 and Vero cells were either infected with MeV ld-MeVami-122 H-EGFP (MeVami-122) at MOI 0.3 or transfected with 1 µg pCG MeVami-122 and harvested 25 – 30 h p.i., when cells were completely in syncytia. Mature and primary miRNA was isolated and analyzed via qPCR in technical triplicates. Relative expression to pCG-derived miRNA is shown. Mean values of n=2 experiments are plotted. Error bars indicate standard deviation.
